# Phylogenetic characterisation of circulating, clinical influenza isolates from Bali, Indonesia: preliminary report from the BaliMEI project

**DOI:** 10.1186/s12879-017-2684-2

**Published:** 2017-08-23

**Authors:** W. Adisasmito, S. N. Budayanti, D. N. Aisyah, T. Gallo Cassarino, J. W. Rudge, S. J. Watson, Z. Kozlakidis, G. J. D. Smith, R. Coker

**Affiliations:** 1 Universitas Indonesia, Depok, Indonesia; 2 Universitas Udayana, Denpasar, Indonesia; 3 Farr Institute of Health Informatics Research, University College London, London, UK; 4 Division of Infection and Immunity, University College London, 222 Euston Road, London, NW1 2DA UK; 5 London School of Hygiene and Tropical Medicine, London, UK; 6 Wellcome Trust Sanger Institute, Hinxton, UK; 7 Duke Global Health Institue, Duke University, Durham, NC USA

**Keywords:** Next generation sequencing, Influenza, Bali, Indonesia, Phylogeny

## Abstract

**Background:**

Human influenza represents a major public health concern, especially in south-east Asia where the risk of emergence and spread of novel influenza viruses is particularly high. The BaliMEI study aims to conduct a five year active surveillance and characterisation of influenza viruses in Bali using an extensive network of participating healthcare facilities.

**Methods:**

Samples were collected during routine diagnostic treatment in healthcare facilities. In addition to standard clinical and molecular methods for influenza typing, next generation sequencing and subsequent de novo genome assembly were performed to investigate the phylogeny of the collected patient samples.

**Results:**

The samples collected are characteristic of the seasonally circulating influenza viruses with indications of phylogenetic links to other samples characterised in neighbouring countries during the same time period.

**Conclusions:**

There were some strong phylogenetic links with sequences from samples collected in geographically proximal regions, with some of the samples from the same time-period resulting to small clusters at the tree-end points. However this work, which is the first of its kind completely performed within Indonesia, supports the view that the circulating seasonal influenza in Bali reflects the strains circulating in geographically neighbouring areas as would be expected to occur within a busy regional transit centre.

**Electronic supplementary material:**

The online version of this article (doi:10.1186/s12879-017-2684-2) contains supplementary material, which is available to authorized users.

## Background

Virus gene sequencing and phylogenetics can be used to study the epidemiological dynamics of rapidly evolving viruses. With complete genome data, it becomes possible to identify and trace individual transmission chains of circulating viruses such as influenza virus within a defined spatiotemporal context. Next generation sequencing (NGS) has been employed in the high-throughput production of complete viral genome data and offers significant opportunities for increasing our understanding of influenza distribution and transmission. To date, NGS has been used on influenza samples as the basis for identification [1–4] and comparison [5, 6] of full influenza genomic data as well as for applications such as profiling quasi-species and lineage [7, 8] and characterising interactions with the host-cell. [9, 10] The Molecular Epidemiology of Influenza A in Bali project (“BaliMEI”) [11] aims to conduct five years of active surveillance and characterisation of influenza viruses in Bali (2010–2015). The project utilises a network of 21 health facilities across all nine regencies of Bali to collect nasopharyngeal swabs from patients presenting with influenza-like illness. Here we report the whole genome sequencing results and phylogenetic analysis of the first 95 influenza samples from the BaliMEI project.

Indonesia is of key strategic importance for influenza surveillance and research, as it continues to report highly pathogenic avian influenza (H5N1) outbreaks in poultry, along with sporadic cases in humans. [12] However, research on the ecology and evolution of influenza viruses in Indonesia has been severely limited. Within Indonesia, the island province of Bali might be a particular hotspot for the mixing of influenza viruses from different geographic regions and host species and potential genomic reassortment thereof, due to high densities and close proximity of humans, poultry and pigs, along with its status as a popular tourist destination with continuous, high numbers of a transient population. [13, 14] Importantly the preliminary results from the BaliMEI study allow us to assess the degree of agreement between epidemiologically and genetically inferred information, understand the extent of mutation observed between genetically clustered cases and to improve estimates of the extent of diversity within the circulating, clinically presented influenza.

## Methods

### Sample collection

The samples were taken during routine diagnostic treatment by hospital physicians between July 2010 and July 2013. All laboratory-confirmed (PCR-positive) samples containing influenza virus from 95 patients were collected through the BaliMEI project protocol under ethics approval number: 41/H2.F10/PPM.00/2010 (University of Indonesia) and 441/Skrt/VI/2010 (Udayana University). These included samples submitted from 21 sentinel health facilities (10 government hospitals and 11 urban health centres) across 8 regencies and one provincial capital city of Denpasar. The 64 samples were obtained from the patients concurrently with epidemiological information through a questionnaire containing sample dates, admission and discharge dates, age, sex, timing of hospital admission and discharge. Patient identifiers were removed prior to the transfer of information to a dedicated, secure Data repository. Laboratory specimen numbers (identifying unique specimens) were retained as these were not interpretable outside the laboratory environment.

### Sample sequencing

RNA was extracted from collected specimens that had been stored at -70 °C temp using the QIAamp Viral RNA Mini Kit. RNA extracts were amplified using a modified eight-segment method [5, 15] and library prepared using the Illumina library preparation Kit Nexterra XT at the biomolecular laboratory at Udayana University. The sequencing used the Ilumina MiSeq platform at Pandu Biosains Laboratory. The average read depth and average genome coverage were recorded across all segments.

### Sequence de novo assembly

Genome assembly and construction of consensus sequences was performed at Indonesia using the Infection response through virus genomics (ICONIC) bioinformatics pipeline for de novo viral sequence assembly [16] developed at University College London (UCL) with phylogenetic analyses to infer transmission performed at the Farr institute of Health Informatics Research. In short, NGS data were subjected to quality control using Trimmomatic 0.33 to remove any primer sequences and trim reads, then reads mapped with SMALT version 0.7.6 (http://www.sanger.ac.uk/science/tools/smalt-0) to the human genome were removed. Quality controlled and filtered read sets were de novo assembled using IVA version 1.0.0 [17]; SAMtools 1.2 [18] and custom scripts were used to create a consensus genome from the assembled fragments (“contigs”). In particular, these scripts utilise BLAST to find the closest matching sequences to the draft segments and use them as templates on which to map unassembled reads with SMALT.

### Phylogenetic analysis

The influenza virus sequences were combined with existing sequences retrieved from the NCBI Influenza Virus Resource [19] that represented the range of genetic diversity worldwide during the same period (Additional file 1). Each genome segment was aligned separately using the MUSCLE aligner [20] provided in MEGA version 6.06. [21] Separate alignments were made for H1N1 and H3N2 sequences. Alignments were then trimmed to coding regions, and sequences covering less than 50% of the coding region were removed. Phylogenetic trees were then inferred under a maximum-likelihood (ML) criterion using RAxML version 7.2.8. [22] Maximum likelihood (ML) trees were estimated for all the eight gene segments using the best-fit general time reversible (GTR) model of nucleotide substitution with a gamma distribution of among-site rate variation (with four rate categories, Γ4) and an SPR branch-swapping search procedure implemented in PhyML [23]. Tree robustness was determined through bootstrap analysis of 1000 sequence pseudoreplicates. Trees were visualized using FigTree version 1.4.2 (http://tree.bio.ed.ac.uk/software/figtree/).

## Results

**Fig. 1:**
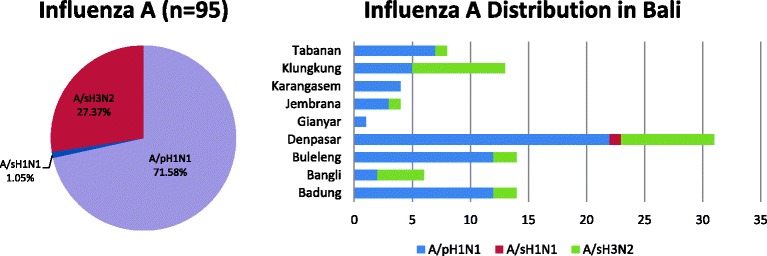
Relative distribution of the influenza A types collected in Bali (2010–2013) shown on the pie chart (left). Relative distribution of the influenza A samples collected in Bali, broken down by geographical origin shown in the bar chart (right)

Among the 95 PCR-positive, patient samples with influenza A, the mean age was 14.65 ± 15.56 years old (range 0.5–40 yo), 60% were male, and 27.4% were submitted from hospital. There were a total of 7 samples collected in 2010, 36 samples in 2011, 25 samples in 2012, and 27 samples in 2013. Influenza due to influenza A viruses mostly occurred in children age 0–4 yo (35.8%), following in children age 5–14 yo (27.4%) and 15–24 yo (12.6%). A summary of these results is shown in Table 1. From the 95 samples, 68 (71.6%) were subtyped as A/H1N1-pdm09, 26 (27.4%) as A/H3N2, and 1 (1.1%) as seasonal A/H1N1 (Fig. 1a). The highest number of samples were collected from Denpasar (32.6%), followed by Badung and Buleleng (14.5% and 14.5% respectively) (Fig. 1b) correlating to their relative population density. There was no correlation observed between the calendar time of sample collection 108 and relative numbers of samples, as all influenza types analysed in the present paper were collected throughout the 2010–2013 period.

**Table 1:** Summary of influenza samples collected among patients presenting with influenza-like illness, categorised by sex, age group and facility type, BaliMEI (2010–2013)

		Influenza A virus						*P* ^†^
		A/H1N1-pdm09		A/H3N2		A/sH1N1		
All patients	95	68	(71.6)	26	(27.4)	1	(1.1)	
Sex								
Female	38	28	(41.2)	10	(38.5)	0	(0.0)	0.69
Male	57	40	(58.8)	16	(61.5)	1	(100.0)	
Age group (y)								
0–4	34	24	(35.3)	10	(38.5)	0	(0.0)	0.36
5–14	26	16	(23.5)	10	(38.5)	0	(0.0)	
15–24	12	10	(14.7)	2	(7.7)	0	(0.0)	
25–34	11	8	(11.8)	2	(7.7)	1	(100.0)	
35–44	6	5	(7.4)	1	(3.8)	0	(0.0)	
45–64	6	5	(7.4)	1	(3.8)	0	(0.0)	
Facility type								
Hospital	26	21	(30.9)	5	(19.2)	0	(0.0)	0.44
Health centre	69	47	(69.1)	21	(80.8)	1	(100.0)

Of the 95 collected, PCR-positive influenza A samples, 62 were of sufficient concentration (Ct < 30) to be further processed through an NGS platform in an attempt to generate more extensive viral genomes with high read-depth coverage (17 samples A/H3N2, 45 samples A/H1N1-pdm09). As a result of the NGS sequencing and the subsequent de novo assembly, 31 complete influenza A genomes were assembled with all 8 genomic segments, while for the remaining samples, there was an average recovery of 5 out of the genomic 8 segments. There was complete correspondence of the influenza A typing between the PCR- and NGS-based methods across all of the samples. Across the samples in which it was possible to build segments, the average read depth was 2443 and the average genome coverage was 80%. In particular, segment 3 had the lowest average depth, 559 reads, and segment 1 the lowest average coverage with 56%; while segment 7 had the highest depth, 6073 reads, and segment 8 the highest coverage with 97%. The genome coverage was calculated against the reference found for each sample segment.

**Fig. 2:**
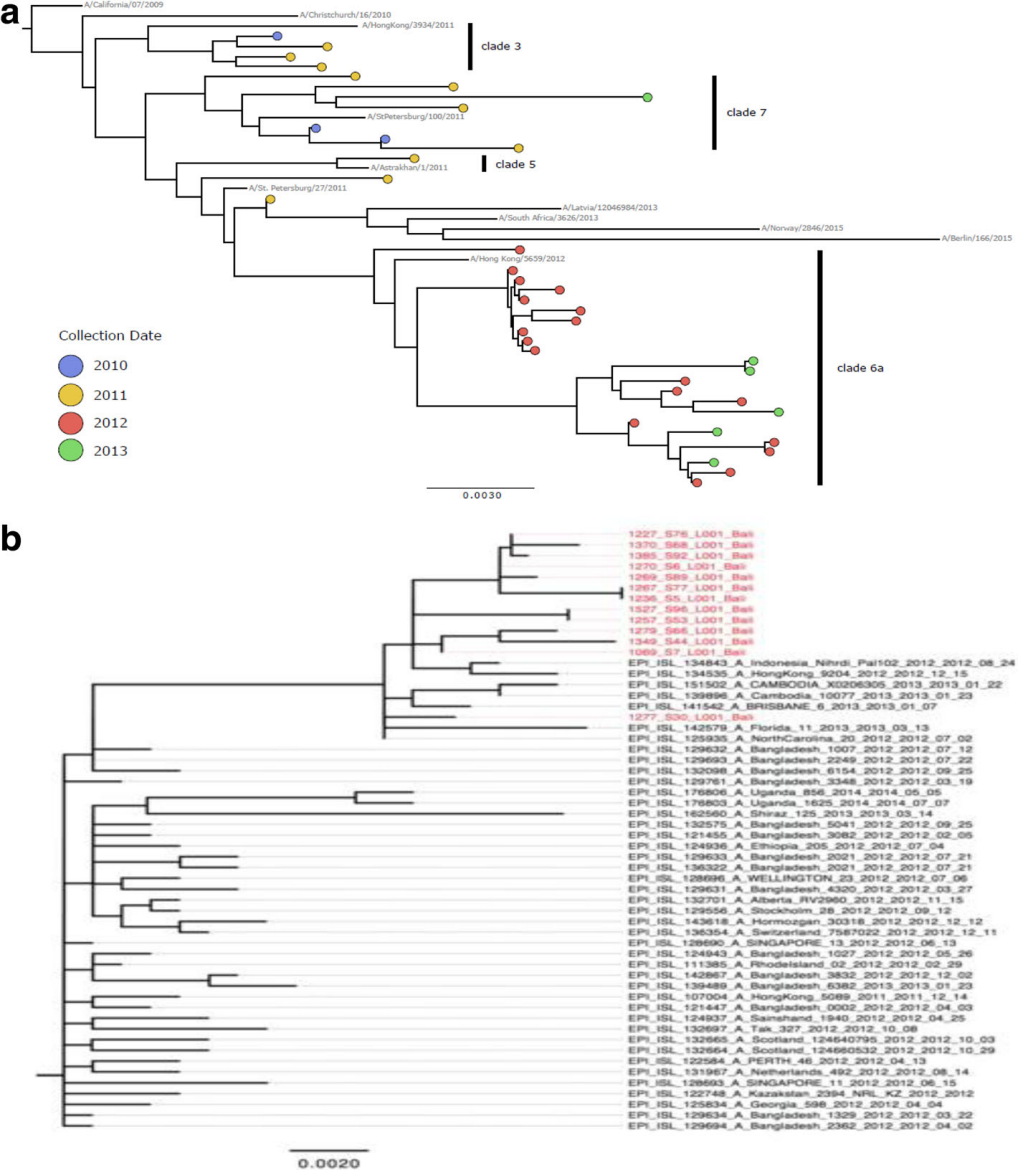
**a** Maximum-likelihood (ML) tree inferred from the nucleotide sequence of the HA gene for the pandemic H1N1 lineage. The tree is rooted on the A/California/07/2009 strain. Reference isolates for each subclade are shown in grey, with isolates from BaliMEI shown as circles, with colours indicating the year of isolation. Subclades containing BaliMEi isolates are highlighted with a black bar and labelled with the corresponding clade name. The scale bar is given in units of substitution per site. **b** Magnification of the Balinese cluster within clade 6a, taken from a higher-resolution ML tree containing globally-collected isolates. BaliMEI isolates are labelled in red. The scale bar is in units of substitutions per site. The details of the samples included in this analysis are available in Additional file 1

According to the phylogenetic analyses all samples analysed were of the influenza type circulating globally at the time of collection and in particular in proximal countries, such as Singapore, Thailand, Australia and Cambodia. Some samples formed small clusters at the phylogenetic tree end-points indicative of a small-scale localised circulation within Bali, and lack of subsequent transmission to other neighbouring countries or localities (Fig. 2 showing H1N1 samples; all H3N2 samples gave identical distribution – data not shown). However the sample number is small to support any further observations based on the phylogeny alone.

## Discussion

The Influenza patient samples analysed above and collected at Bali, Indonesia, are considered typical of the background seasonal influenza incidence that is ever present in the area. There were no statistically significant differences in the distribution of the samples across the sexes, months of the years or localities. There was a slight tendency towards younger age-groups (0 – 14yo, 46% of samples), where the need for hospitalisation is perhaps more acute than in older age groups, while more densely populated areas and built up areas provided larger numbers of samples in the collection. However a larger sample size would be needed to confirm this observation. The influenza patient samples were typed independently by PCR under routine hospital protocols and by NGS methods; the resulting absolute correlation of the typing is supportive of the NGS-based results. The sequence depth from the influenza samples is adequate for the de novo genomic assembly; however the depth variability across the fragments does not allow the identification of minority variants in a genome-wide approach. The variable depth of the obtained sequences is most likely a reflection of the collection approach where PCR-positive samples were collected during routine diagnostic treatment without a preselection on the Ct value of the viral sample. Quantitative sample pre-selection might result in better NGS output with regards to sequence read-depth but also introduce potential sample bias in the collection. There were no complex reassortant viruses nor any oseltamivir-resistance markers amongst the 62 NGS samples analysed.

The phylogenetic analysis supports the view of these samples being representative of seasonal, circulating Influenza amongst the population in Bali. There were, as expected, strong phylogenetic links with sequences from samples collected in geographically proximal regions, with some of the samples from the same time-period resulting to small clusters at the tree-end points. However more samples would need to be analysed before further claims can be made regarding the influenza transmission in Indonesia from either a local or international perspective. The current paper represents the first ever attempt to utilise NGS technology to characterise clinically relevant samples from Bali, Indonesia as part of the Bali-MEI project. Importantly the current first report reflects work that has been almost entirely taken place within the country as testament to current capacity building efforts and future prospects for even further and more detailed influenza surveillance within Indonesia.

## Conclusion

We describe the preliminary results from a five year project on the surveillance and characterization of seasonal influenza samples from healthcare facilities in Bali, Indonesia. This work is the first of its kind completely performed within Indonesia, and can be used as the blueprint for future national molecular surveillance projects. The results support the view that the circulating seasonal influenza in Bali reflects the strains circulating in geographically neighbouring areas as would be expected to occur within a busy regional transit centre.

## Additional files

Additional file 1: Sequence IDs from the GISAID database. Sequences downloaded from the GISAID database representing the range of influenza genetic diversity worldwide during the same period that the BaliMEI study was conducted (XLS 876 kb)

Additional file 2: Sequence IDs from the GISAID database. The IDs of the BaliMEI influenza sequences uploaded to the GISAID database (XLS 79 kb)

## Abbreviations

NGS: Next Generation Sequencing
BaliMEI: Molecular Epidemiology of Influenza in Bali, Indonesia
PCR: Polymerase Chain Reaction
ICONIC: Infection response through virus genomics
UCL: University College London
NCBI: National Centre for Biotechnology Information.
